# Effect of Zirconium Dioxide Nanoparticles on Glutathione Peroxidase Enzyme in PC12 and N2a Cell Lines

**Published:** 2014

**Authors:** Elham Asadpour, Hamid Reza Sadeghnia, Ahmad Ghorbani, Mohammad Taher Boroushaki

**Affiliations:** a *Department of Pharmacology, Faculty of Medicine, Mashhad University of Medical Sciences, Mashhad, Iran.*^*. *^; b *Neurocognitive Research Center, Faculty of Medicine, Mashhad University of Medical Sciences, Mashhad, Iran.*; c *Pharmacological Research Center of Medicinal Plants, Faculty of Medicine, Mashhad University of Medical Sciences, Mashhad, Iran.*

**Keywords:** Zirconium dioxide nanoparticles; PC12 cell line; N2a cell line; Oxidative stress; Glutathione peroxidase enzyme

## Abstract

Today, special attention is paid to the use of zirconium dioxide nanoparticle (nano-ZrO_2_), a neutral bioceramic metal, particularly for drug and gene delivery in medicine. However, there are some reports implying that use of nano-ZrO_2_ is associated with cytotoxic effects like inhibiting the cell proliferation, DNA damage and apoptosis. In the present study, we examined whether nano-ZrO_2_ alters cell viability and glutathione peroxidase (GPx) activity in two neuronal cell lines.

The PC12 and N2a cells were cultured in the absence or presence of varying concentrations (31.25-2000 µg/mL) of nano-ZrO_2_ for 12, 24 or 48 h. The cell viability was evaluated using 3-(4,5-dimethylthiazol-2-yl)-5-(3-carboxymethoxyphenyl)-2-(4-sulfophenyl)-2H-tetrazolium (MTS) assay and GPx activity was determined by quantifying the rate of oxidation of the reduced glutathione to the oxidized glutathione.

Nano-ZrO2 caused a significant reduction in cell viability and GPx activity after 12, 24 and 48 h, as compared with control group. These effects were concentration dependent and started from 250 µg/mL.

The present study demonstrated that nano-ZrO_2_, at concentrations of > 250 µg/mL, has antiproliferative effects via reducing the cell defense mechanism against oxidative stress.

## Introduction

Zirconium dioxide (ZrO_2_), an essential trace metal, is a silvery, lustrous, strong transition metal which is very similar to titanium and hafnium (1-2). In recent years, usage of Zirconium dioxide nanoparticles (nano-ZrO_2_) is rapidly growing in biological fields. they are widely used as drug delivery carriers for some medicines like itraconazole, penicillin, alendronate and zoledronate (-); as gene delivery vehicles with target specificity for some tissues (6-7); for improving the properties of traditional bone cements in orthopedia (8); and some other purposes such as production of poisons like parathion (9) or nerve agents (10).

Although ZrO_2_ is a neutral bioceramic metal, but it is propounded that the introduction of these materials into clinical practice may be associated with some short- and long-term risks in living systems. For example, a significant DNA damage in human T-cells (11), an induction of apoptosis in human mesenchymal stem cells (12), and inhibition of cell proliferation in human mesothelioma (MSTO-211H) and rodent fibroblast cell lines (13) are reported after exposure to zirconium.

Increased levels of reactive oxygen species (ROS) are involved in DNA damage and apoptosis because they damage cellular macromolecules including lipids, proteins and nucleic acids (14). Neurons are especially sensitive to the oxidative stress due to their high rate of oxidative metabolic activity and having relatively low amounts of endogenous antioxidants (15-16). As a result of high ATP demand of the brain, O_2_ is consumed rapidly and free radicals are a constant production in this tissue.

Tissue and cellular damage can be resulted from extensive ROS production in inflammatory disease (17). Superoxide (O_2_) and its product hydrogen peroxide (H_2_O_2_) can even produce more reactive species like hydroxyl radical, hypochlorous acid and singlet oxygen which can damage the components of extracellular matrix (18-19). Moreover, localized effect of ROS will continue to the production of extra cytokine and more damage (20). Cell signaling episodes can be initiated in macrophages by H_2_O_2 _which lead to the activation of transcriptional factor NF-κB (19, 21). 

Studies have shown that, in turn, cell can defense itself with multiple antioxidant enzymes including superoxide dismutase (SOD), Glutathione Peroxidase (GPx) and catalase (CAT) (22-23). The GPx enzyme belongs to a family of selenoproteins whose function is to catalyze the reduction of various peroxides (24). GSH-Px acts as the second line of defense by converting peroxide into water and molecular form of oxygen. Especially, in mammalian cells it plays a critical role to protect them from oxidative stress (25-26). 

An imbalance between the productions of free radicals and the cell ability for detoxifying these radicals is involved in molecular mechanism of neurotoxicity (27-28). 

Since, the cytotoxic effects of nano-ZrO_2_ has not been investigated before, this study was designed to elucidate the effect of these nanoparticles on viability and GPx enzyme activity of N2a and PC12 cells.

## Experimental

*Cell lines and reagents*

Commercial nano-ZrO_2_ (<100 nm in average size) were purchased from Sigma-Aldrich (St Louis, MO). PC12 and N2a cell lines, derived from rat pheochromocytoma and mouse neuroblastoma, respectively, were purchased from Pasteur Institute (Tehran, Iran). High glucose (4.5 g/L) Dulbecco’s Modified Eagles Medium (DMEM) and fetal calf serum were purchased from Gibco (Carlsbad, CA). 3-(4,5-dimethylthiazol-2-yl)-5-(3-carboxymethoxyphenyl)-2-(4-sulfophenyl)-2H-tetrazolium (MTS) was obtained from Promega (Madison, WI, USA). Glutathione Peroxidase (GPx) Assay kit was purchased from Northwest Life Science Specialties, LLC (Vancouver, WA USA).

*Dispersion and characterization of the zirconia nanoparticles*

Nano-ZrO_2_ was suspended in media and dispersed by an ultrasonic bath for 60 min. Then the size of nanoparticles in suspension was characterized by Transmission Electron Microscopy (TEM, LEO 912AB, Germany), using one drop of sample on a carbocoated grid and high voltage (120K.V.) imagining TEM.

*Cell culture and nanoparticle treatment*

The cells were maintained in Dulbecco's modified Eagle's medium (DMEM) containing 10% fetal calf serum, penicillin 100 IU/mL, and streptomycin 100 µg/mL. They were grown and maintained in 75 cm^2^ cell culture flasks at 37 ^o^C in a 5% CO_2_ humidified incubator. The test suspension of nano-ZrO_2_ was prepared using the culture media and dispersed for 20 min by using a sonicator (Bandelin electronic, DT 510, Germany) to prevent aggregation. The cells were treated with various concentrations of nanoparticles according to the time schedule. 

*Cell viability assay*

The cell viability assay is based on the reduction of novel tetrazolium compound (MTS) by mitochondrial dehydrogenase in metabolically active cells to the water-soluble formazan (29). About 10^4^ cells were seeded in each well of a 96-microwell plate and treated with various concentrations (0-2000 µg/mL) of nanoparticle suspension for 12, 24 and 48 h. At the end of the treatment, the MTS reagent, which is composed of the novel tetrazolium compound and an electron coupling reagent phenazine ethosulfate, was added to the cell media. After 3 h, cell viability was determined by measuring the optical absorbance at 490 nm using an ELISA microplate reader (Awareness, Palm City, FL, USA). The cytotoxicity of nano-ZrO_2_ was calculated using Graph Pad Software (Graph Pad prism 5 software) and presented as mean ± SEM of three independent experiments with three replicates for each concentration.

*Determination of glutathione peroxidase activity*

Glutathione peroxidase activity was determined using a sensitive kit produced based on the method of Paglia and Valentine (30). In this method, glutathione (GSH) is oxidized to form oxidized glutathione (GSSG) which is then reduced by glutathione reductase (GR) and b-nicotinamide adenine dinucleotide phosphate (NADPH) forming NADP+ (resulting in decreased absorbance at 340 nm) and recycling the GSH. Because GPx is limiting, the decrease in the absorbance is directly proportional to the GPx concentration.

Cells were seeded in 6-well tissue culture plates with 10^6^ cells per mL media. After allowing the cells to be seeded for 24 h, nanoparticle suspension with different concentration (2000, 1000, 250, 62.5, 31.25 µg/mL) were added to each well for 12, 24 and 48 h. Then, Cells were washed with PBS, scraped and homogenized in 20 mM PBS containing 0.5 mM butylated hydroxyltoluene to prevent sample further oxidation. The homogenate was centrifuged at 3,000 g for 20 min at 4°C, and the supernatant was used for this assay according to the manufacturer’s instructions. The amount of protein in cell extract was determined by the Bradford method. Fluorescence was recorded in a FLUO-star galaxy fluorescence plate reader (Perkin Elmer 2030, Multi label reader, Finland) at 340 nm wavelength. All experiments were carried out in triplicate. The enzyme activity was expressed as U/mg Protein.

*Statistical analysis*

The results are presented as the mean ± SEM. The values were compared using the one-way analysis of variance (ANOVA) followed by Tukey’s post tests for multiple comparisons. The p<0.05 was considered to be statistically significant.

## Results

*Characterization of nano-ZrO*_2_

**Figure 1:**
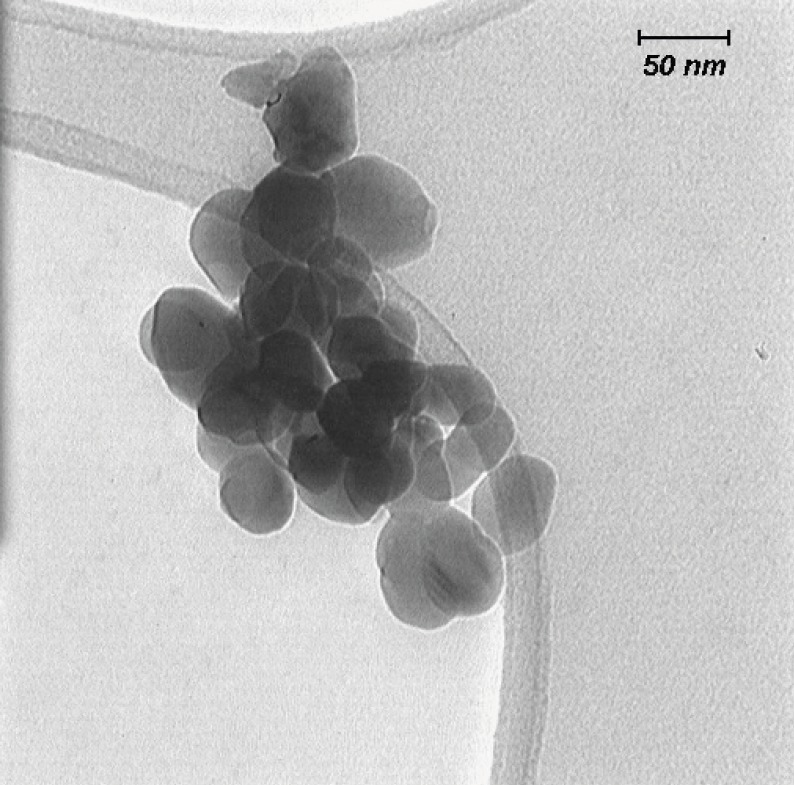
Transmission Electron Microscopy (TEM) micrograph of nanoscale zirconium dioxide suspended in media and dispersed by an ultrasonic bath

The size of the nano-ZrO_2_ was found to be less than 100 nm as shown in the images of Transmission Electron Microscopy (Figure 1). 

*Effect of* *nano-ZrO*_2_ *on cell viability*

**Figure 2:**
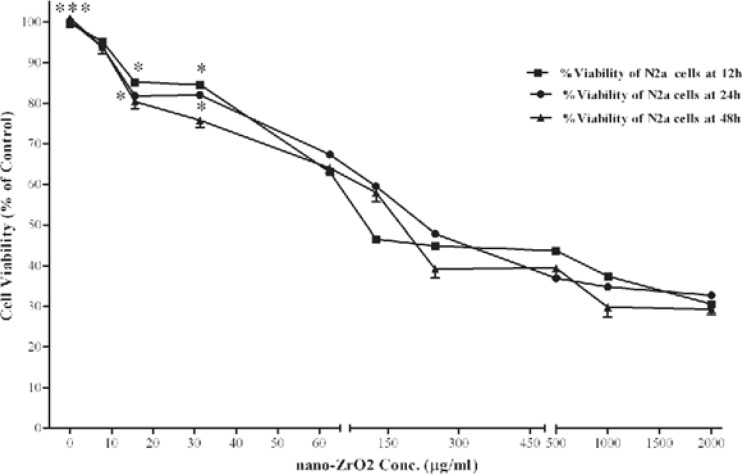
Effects of nano-ZrO2 on N2a cell viability. Cells were treated with increasing concentrations of zirconia for 12, 24 and 48 h. The cell viability (quantified by MTS assay) is shown and discussed as percentage of control group (zirconia 0 µg/mL). Mean and SEM of three independent experiments are shown. One way ANOVA analysis shows significant difference between the data which were investigated in each time. ***p<0.001 versus all concentration more than 15.6 µg/mL in all 3 exposure time and *P<0.05 versus control group

The effect of nano-ZrO_2_ on viability of N2a cells are shown in Figure 2. After 12 h incubation, cell viability was significantly decreased at concentrations of ≥15.6 µg/mL. As compared with untreated cells (100 ± 9.3 %), the nano-ZrO_2_ at 2000 µg/mL decreased the cell viability to 32.76 ± 2.8 (*P *< 0.001), which was lowest viability amount among all the concentrations. Similarly, exposure of the N2a cells to the nanoparticles for 24 and 48 h showed a concentration-dependent decrease in cell viability. Again the cytotoxic effect was observed at concentrations more than 15.6 µg/mL (*P *< 0.05 and P<0.001) after 24 and 48 h exposure, respectively. No significant difference in cell viability was found between the three incubation times.

**Figure 3:**
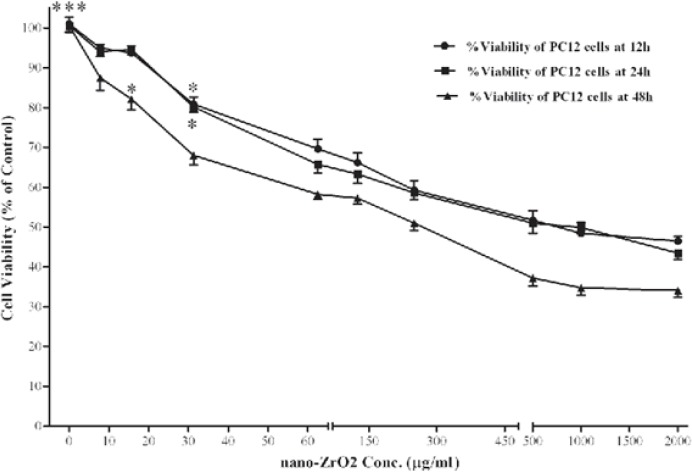
Effects of nano-ZrO2 on PC12 cell viability. Cells were treated with increasing concentrations of zirconia for 12, 24 and 48 h. The cell viability (quantified by MTS assay) is shown and discussed as percentage of control group (zirconia 0 µg/mL). Mean and SEM of three independent experiments are shown. One way ANOVA analysis shows significant difference between the data which were investigated in each time. ***p<0.001 versus all concentration more than 31.25 µg/mL in all 3 exposure time and *P<0.05 versus control group.

Figure 3 shows the effect of nano-ZrO_2_ on viability of PC12 cells. After 12 h, the level of viability in the cells exposed to 31.25, 62.5, 125, 250, 500, 1000 and 2000 µg/mL of nano-ZrO_2_ was 80.90 ± 5.1, 69.66 ± 7.2, 66.23 ± 7.0, 69.29 ± 7.1, 51.75 ± 6.9, 48.45 ± 7.9, and 46.46 ± 3.5 %, respectively, which at concentrations more than 31.25 µg/mL was significantly (*P* < 0.05) lower than that of untreated cells (100 ± 5.2 %). Approximately, the same level of viability was observed when incubation time extended to 24 h. However, 48 h incubation with nano-ZrO_2 _leads to more reduction of cell surviving, so that, its cytotoxicity was started at concentrations of ≥15.6 µg/mL (*P *< 0.05).

*Effect of* *nano-ZrO*_2_ *on glutathione peroxidase activity*

**Figure 4:**
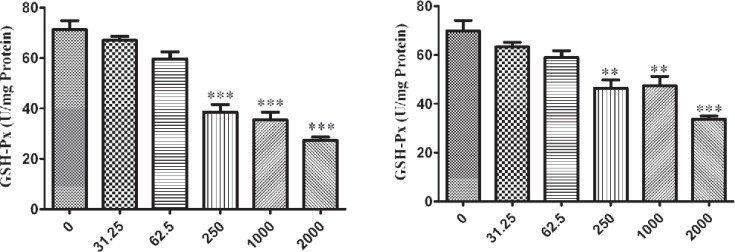
Effects of nano-ZrO2 on glutathione peroxidase activity (GPx), in A: N2a cell line, B: PCl2 cell line. Cells were treated with different concentrations of zirconia for 12 hours. The GPx concentration is normalized to the protein concentration and the activity of enzyme was expressed as unit/mg protein. One way ANOVA analysis show significant difference between the data which were investigated *p<0.05 **p<0.01 and ***p<0.001, as compared with control group

**Figure 5:**
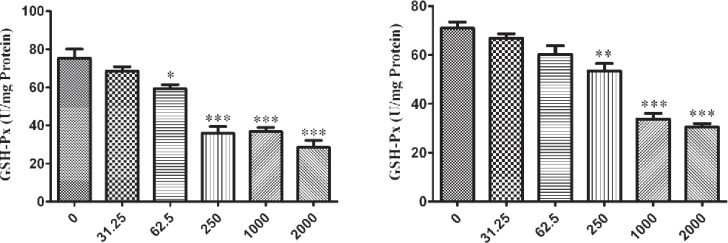
Effects of nano-ZrO2 on glutathione peroxidase activity (GPx), in A: N2a cell line, B: PCl2 cell line. Cells were treated with different concentrations of zirconia for 24 hours. The GPx concentration is normalized to the protein concentration and the activity of enzyme was expressed as unit/mg protein. One way ANOVA analysis show significant difference between the data which were investigated *p<0.05 **p<0.01 and ***p<0.001, as compared with control group

**Figure 6:**
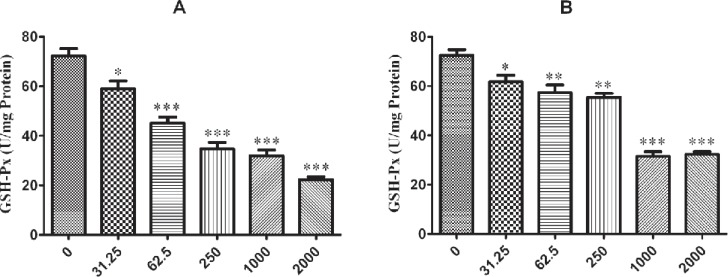
Effects of nano-ZrO2 on glutathione peroxidase activity (GPx), in A: N2a cell line, B: PC12 cell line. Cells were treated with different concentrations of zirconia for 48 hours. The GPx concentration is normalized to the protein concentration and the activity of enzyme was expressed as unit/mg protein. One way ANOVA analysis show significant difference between the data which were investigated *p<0.05 **p<0.01 and ***p<0.001, as compared with control group

The influence of nano-ZrO_2 _on GPx enzyme is determined with decreasing its activity comparing to the untreated group which this reduction is started from 250 µg/mL (p < 0.001) after 12 h exposure of N2a cell line (Figure 4A) and it is distributed to lower concentration via rising the duration of exposure time up to 24 h and 48 h which are 62.5 µg/mL and 31.25 µg/mL (p < 0.05) (Figure 5A and 6A), respectively. 

For PC12 cell line, there is almost the same condition. In this case the level of enzyme activity is decreasing from concentration 250 µg/mL (p < 0.01) after 12 h and 24 h exposure (Figure 4B and 5B) and from concentration 31.25 µg/mL (p < 0.05) after 48 h incubation with nano-ZrO_2_ (Figure 6B).

## Discussion

Considering the increased usage of zirconia nanoparticles in many aspects of biological field, more attention should be considered to their safety or toxicity. The particles can enter the body via inhalation, skin penetration, ingestion or via metallic implant corrosion (31-33). It has been well documented that zirconium dioxide can induce cell cytotoxicity by its own. For instance, Caicedo and his colleagues show that zirconium ion at concentrations more than 5 mM can induce apoptosis in T-Helper Jurkat Cells (11). Moreover, another study showed that zirconia with a particle size around 0.6 µm induces apoptosis through increase of TNF-α release in J744 macrophage cells (34-35).

In this work for the first time we showed that nano-ZrO_2 _particles have antiproliferative effect on N2a and PC12 cell cells. These neuronal cell lines have extensively used in researches to test possible neurotoxic or neuroprotective effects of compounds (36-38). Assessment of nano-ZrO_2 _cytotoxicity through the MTS assay revealed a dose dependent toxic effect on the neuronal cell viability in a concentration range of 31 to 2000 µg/mL. On the other hand, this effect was not changed while incubation time increased, which suggests that the peak of nano-ZrO_2_ cytotoxicity occurs during first 12 h exposure. 

The correlation between oxidative stress and decrease of cell viability has been well documented (39-40). The unwanted effects of oxidative stress is resulted from increasing the lipid peroxidation, DNA damage and apoptosis induction (41). The mitochondrial thiol proteins, in particular GPx, have a critical role in protecting the cell against redox signaling and cell death programming (42). Here we showed that nano-ZrO2 decrease GPx activity and therefore oxidative stress may be, in part, involved in the cytotoxicity effect of this nanoparticle. Our finding is in accordance with what olmedo and his colleagues revealed in 2011 about the effect of zirconia on oxidant–antioxidant balance in tissues (43). Also it was previously reported that submicron Zirconia increases the release of superoxide (O2^-^) and nitric oxide by Human THP-1 Macrophages (19). Taken together, direct interaction of nano-ZrO_2 _with cell membrane and/or accumulation of free radicals such as HO2*•*, O2*•*, HO*•* and carbon-centered radicals as a result of GPx activity decrease, can initiate oxidative damage and induce cell apoptosis (40, 44-46).

In conclusion, our study showed that nano-ZrO_2 _at concentrations more than 250 µg/mL has neurotoxic effects through increasing the oxidative stress and this should be considered for therapeutic use. 
